# Unroofing site-specific α-synuclein–lipid interactions at the plasma membrane

**DOI:** 10.1073/pnas.2006291117

**Published:** 2020-07-27

**Authors:** Upneet Kaur, Jennifer C. Lee

**Affiliations:** ^a^Laboratory of Protein Conformation and Dynamics, Biochemistry and Biophysics Center, National Heart, Lung, and Blood Institute, Bethesda, MD 20892

**Keywords:** unroofed cells, GM1, NBD, fluorescence lifetime

## Abstract

Parkinson’s disease is associated with α-synuclein (α-syn), a cytosolic protein enriched in presynaptic terminals. The biological function of α-syn remains elusive; however, increasing evidence suggests that the protein is involved in the regulation of synaptic vesicle fusion, signifying the importance of α-syn–lipid interactions. We show that α-syn preferentially binds to GM1-rich, liquid-ordered lipid domains on cytoplasmic membranes by using unroofed cells, which encapsulates lipid complexity and cellular topology. Moreover, proteins (Rab3a, syntaxin-1A, and VAMP2) involved in exocytosis also localize with α-syn, supporting its proposed functional role in exocytosis. To investigate how these lipid/protein interactions influence α-syn at the residue level, α-syn was derivatized with an environmentally sensitive fluorophore (7-nitrobenz-2-oxa-1,3-diazol-4-yl [NBD]) at different N- and C-terminal sites. Measurements of NBD fluorescence lifetime distributions reveal that α-syn adopts a multitude of membrane-bound conformations, which were not recapitulated in simple micelle or vesicle models, indicating an exquisite sensitivity of the protein to the complex lipid environment. Interestingly, these data also suggest the participation of the C terminus in membrane localization, which is generally overlooked and thus emphasize the need to use cellularly derived and biologically relevant membranes for biophysical characterization. Collectively, our results demonstrate that α-syn is more conformationally dynamic at the membrane interface than previously appreciated, which may be important for both its physiological and pathological functions.

## Significance

α-Synuclein is a neuronal protein with an ill-defined biological function that is central to Parkinson’s disease etiology. While considered to be involved in exocytosis, how α-synuclein facilitates synaptic vesicle fusion and release remains an open question. To address this, we investigated α-synuclein–lipid interactions at the plasma membrane through the technique of cellular unroofing, which uncovers an intact basal membrane. We conclusively show that α-synuclein is recruited to exocytic sites, preferring liquid-ordered lipid domains. Importantly, heterogeneous populations of α-synuclein conformers are revealed by measurements of fluorescence lifetime distributions, which are not adequately described by current models of α-synuclein structures. Membrane-bound α-synuclein is conformationally dynamic, exquisitely sensitive to lipid/protein composition, enabling the protein to carry out its function.

α-Synuclein (α-syn), a widely studied neuronal protein involved in the pathology of Parkinson’s disease, remains an enigma because its physiological functions are not well understood (1). α-Syn is concentrated in presynaptic terminals, where it has been proposed to be involved in synaptic vesicle docking, fusion, clustering, and homeostasis (2, 3). As these functions occur on the lipid membrane surface, the ability for α-syn to interact with lipids is essential (4).

Upon membrane binding, α-syn adopts an α-helical structure (5–7), whereas cytosolic α-syn is intrinsically disordered (8). The lipid-binding domain consists of seven imperfect 11-residue repeats that form an amphipathic helix upon membrane association in vitro (9, 10). When membrane-bound, a dynamic equilibrium exists between an elongated helix, encompassing the first 100 residues, and a short N-terminal helix (residues 1 to 25) with a weakly associated unstructured region (residues 26 to 98) (11). While the acidic C-terminal region (residues 100 to 140) remains in solution for both conformations, there is evidence to suggest a functional role for the C terminus as it can bind calcium ions and mediate interactions with SNARE proteins such as VAMP2 (12–14); however, the subcellular spatial context in which this interaction occurs remains unknown. Additionally, a flexible linker region (residues 38 to 44) can be induced by membrane curvature, which gives rise to a broken helix (15–17). While the conformational plasticity of membrane-bound α-syn is proposed to be biologically important (18), direct observation of α-syn conformational heterogeneity at the residue level has yet to be realized on a cellular membrane.

α-Syn–lipid interactions are modulated by lipid composition, membrane fluidity, and curvature (19). Upon binding, α-syn can influence lipid packing within the bilayer (20), induce clustering of vesicles (13), and generate formation of curved lipid structures, such as tubules (21–23) and nanodiscs (24, 25). A large body of work has shown that binding and folding of α-syn are most prevalent when negatively charged, small unilamellar vesicles are present (7, 26–28). Contrary to anionic lipids (29), the protein exhibits the unusual behavior of preferring ordered gel phases of zwitterionic phospholipids in vitro (30, 31), driven by its enhanced affinity for lipid-packing defects (30). This observation is pertinent due to the similarity in the lipid composition to those found in cellular liquid-ordered lipid domains (32), enriched in cholesterol and sphingolipids associated with exocytic sites. However, it remains to be established whether α-syn is found in liquid-ordered lipid domains in vivo (33).

Here, we sought to develop a molecular understanding of α-syn–lipid interactions on cytosolic membranes as related to its biological function. We took advantage of a method called cellular unroofing, which disrupts the upper plasma membrane by brief pulses of ultrasonic vibration, releasing soluble cytoplasmic components, and leaves behind an intact basal membrane (*SI Appendix*, Fig. S1). Using this methodology, numerous vesicular structures and native membrane–protein complexes are preserved along with the cytoskeletal network, achieving the relevant membrane topology of the inner leaflet of the plasma membrane (34). To investigate direct α-syn–lipid interactions on unroofed cells, we employed site-specific Cys variants (V26C, V40C, and Y136C), which were derivatized with an environmentally sensitive fluorophore, 7-nitrobenz-2-oxa-1,3-diazol-4-yl (NBD) (35). NBD serves as a direct membrane-binding reporter, exhibiting a fluorescence spectral shift with substantially enhanced intensity and lifetime when it is transferred to a hydrophobic lipid environment from a water-exposed state (36–38). In addition, since NBD is weakly emissive in water, there is little contribution from the unbound proteins, minimizing fluorescence background.

We find that α-syn binds preferentially to GM1-rich, liquid-ordered lipid domains, adopting a multitude of conformers at exocytic sites, where SNARE proteins such as VAMP2 and syntaxin-1A reside. Conformational elasticity of membrane-associated α-syn was directly observed on cytoplasmic membranes using NBD lifetime imaging. Interestingly, both N and C termini are involved in membrane localization, experiencing local conformational heterogeneity, which is difficult to reconcile with current structural models of membrane-bound α-syn. By using intact cellular membranes, molecular insights were gained on the dynamic nature of membrane-associated α-synuclein, in which the protein adapts to complex lipid and protein environments in order to carry out its function at exocytic sites.

## Results

### Unroofed SK-MEL-28 Cells, a Biologically Relevant Membrane Surface.

**Fig. 1.**
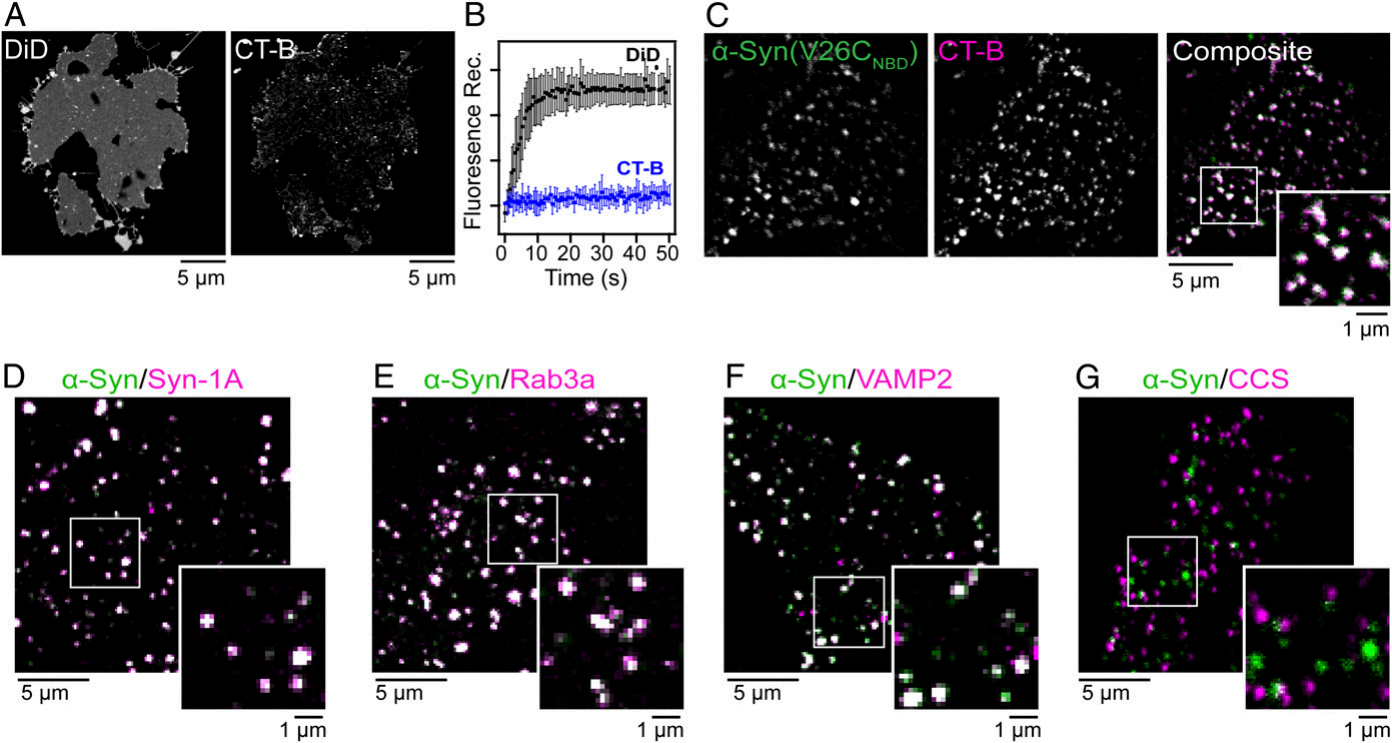
α-Syn localizes to liquid-ordered lipid domains and exocytic sites on unroofed cells. (*A*) Confocal fluorescence images of DiD- and CT-B–stained unroofed SK-MEL-28 cells. (*B*) Fluorescence recovery traces of DiD (black) and CT-B (blue) after photobleaching. Data are given as mean ± SD (*n* = 10). See *SI Appendix*, Fig. S2 for representative individual region of interest data. Composite confocal fluorescence images of V26C_NBD_–α-syn (green) with (*C*) CT-B (magenta; PCC = 0.56), (*D*) syntaxin-1A (magenta; PCC = 0.77), (*E*) Rab3a (magenta; PCC = 0.88), (*F*) VAMP2 (magenta; PCC = 0.7), and (*G*) clathrin-coated structures (CCS) (magenta; PCC = 0.14). Colocalized areas appear white in the composite images. Expanded views are also shown. Scale bars are as indicated. Additional images can be found in *SI Appendix*, Figs. S4 and S6. Pixel plots for the colocalization analysis can be found in *SI Appendix* Fig. S7.

We chose a human melanoma cell line (SK-MEL-28) as a relevant model because it represents a native cellular environment of α-syn as there are high levels of the endogenous protein (39). Moreover, melanocytes are derived from the neuronal crest, sharing many proteins with neuronal cells including those involved in synaptic vesicle homeostasis (40). Representative confocal fluorescence images of an unroofed cell stained with DiD and cholera toxin B (CT-B) are shown in Fig. 1. The lipophilic DiD exhibited a diffusive staining behavior, indicative of an intact phospholipid bilayer (Fig. 1 *A*, *Left*), whereas fluorescently labeled CT-B, a protein that binds to GM1, a ganglioside enriched in lipid rafts (41, 42), showed numerous puncta distributed across the unroofed cell with a low degree of labeling in the surrounding membrane (Fig. 1 *A*, *Right*).

To confirm that the CT-B puncta represented liquid-ordered lipid domains, we performed fluorescence recovery after photobleaching experiments to evaluate fluorophore mobility in the membrane (Fig. 1*B* and *SI Appendix*, Fig. S2). After photobleaching, there was negligible fluorescence intensity recovery observed for regions labeled with CT-B, confirming that these are rigid structures. In contrast, fluorescence intensity fully recovered for regions stained with DiD after 10 s, consistent with a fluid environment as previously reported for unroofed cells (43). This platform retains native-like properties of the inner leaflet of the plasma membrane, composed of fluid and ordered lipids.

### α-Syn Is Localized to Liquid-Ordered Lipid Domains on Unroofed Cells.

To evaluate α-syn binding to unroofed cells, we first added the most N-terminal NBD-labeled protein at position 26 (V26C_NBD_, 1 µM) due to the strong affinity of the N terminus to bind to lipid membranes (44–46). We note that all proteins herein were expressed as the N-terminally acetylated form as this is a ubiquitous posttranslational modification of α-syn (47). After 5 min of incubation, α-syn is seen in a punctate distribution (Fig. 1*C*), similar to that of the CT-B staining pattern (Fig. 1*A*) with minimal binding to the surrounding membrane areas that are stained by DiD (*SI Appendix*, Fig. S3). As a negative control, we confirmed that the NBD fluorophore did not exhibit an intrinsic affinity for unroofed cells (*SI Appendix*, Fig. S3). A Pearson correlation coefficient (PCC) of 0.56 was found for α-syn colocalization with CT-B, indicating that α-syn prefers liquid-ordered lipid domains (Fig. 1*C* and *SI Appendix*, Fig. S4*A*). This preference was verified for endogenous α-syn (*SI Appendix*, Fig. S5*A*). Next, we showed that α-syn also is found in specialized membrane microdomains enriched in gangliosides (e.g., GM1 and GM3) such as caveolae (48, 49). Utilizing an antibody for caveolin-1, a scaffolding protein of caveolae (49), again a high degree of colocalization (PCC = 0.60; *SI Appendix*, Fig. S4*B*) was observed, supporting the finding with CT-B. As a control, a C-terminal Cys-variant (Y136C) labeled with DyLight488 exhibited identical behavior (*SI Appendix*, Fig. S4 *C* and *D*), verifying that the α-syn colocalization pattern on unroofed cells was independent of fluorophore type and labeling position.

### α-Syn Is Localized to Exocytic Sites on Unroofed Cells.

Since α-syn is proposed to be involved in exocytosis, we tested for colocalization of α-syn (V26C_NBD_, 1 µM) with SNARE proteins, syntaxin-1A (target plasma membrane-associated) and VAMP2 (vesicle-associated) as well as a key exocytosis regulatory protein, Rab3a (50). Colocalization analysis of α-syn with syntaxin-1A (Fig. 1*D*), Rab3a (Fig. 1*E*), and VAMP2 (Fig. 1*F*) resulted in PCC values of 0.77, 0.88, and 0.70, respectively. Endogenous α-syn colocalization at exocytic sites was also verified (*SI Appendix*, Fig. S5*B*). The lower PCC value observed for VAMP2 in comparison to Rab3a and syntaxin-1A is because some fraction of VAMP2 is cargo material within clathrin-coated structures (CCS) (51), where we observe no colocalization of α-syn (Fig. 1*G*; PCC = 0.14). The high degree of colocalization exhibited by α-syn with proteins involved in exocytosis strongly supports that α-syn is recruited to regions involved in exocytic vesicle fusion. Because exocytic regions are enriched in cholesterol, GM1, and PtdIns(4,5)P_2_ (52), these colocalization results are consistent with that of CT-B, a specific marker for GM1.

### Fluorescence Imaging Spectroscopy Confirms Direct α-Syn–Lipid Interactions.

**Fig. 2.**
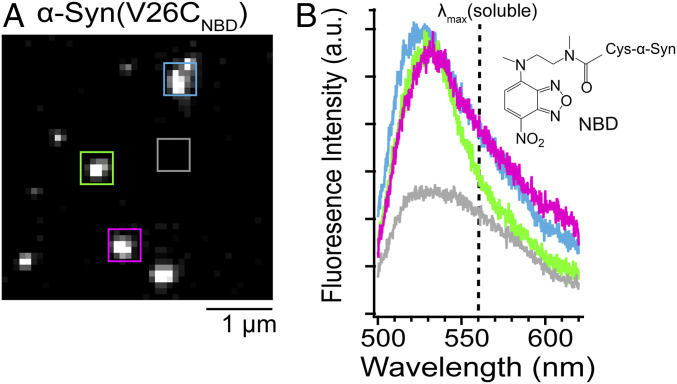
Spatially resolved fluorescence spectra of α-syn bound to unroofed cells. (*A*) Wide-field fluorescence image of V26C_NBD_-α-syn on unroofed SK-MEL-28 cells. Scale bar is as indicated. (*B*) Fluorescence spectra taken at spatial locations indicated by the colored boxes in *A*. The gray box indicates a background spectrum. For comparison, the dashed line represents the emission maximum (λ_max_ = 555 nm) of V26C_NBD_–α-syn α-syn in solution. (*Inset*) Chemical structure of NBD. See *SI Appendix*, Fig. S8 for additional images and spectra.

While fluorescence intensity-based images are spatially informative, they do not conclusively show direct protein–lipid interactions. Thus, we turned to fluorescence imaging spectroscopy to obtain emission spectral information at defined locations. Coupling a spectrograph to an inverted microscope, NBD fluorescence spectra of individual α-syn puncta on unroofed cells were measured (Fig. 2 and *SI Appendix*, Fig. S8). As expected, a large spectral blue shift (emission maxima [λ_max_] = 527 ± 4 nm) was observed compared to that of a water-exposed NBD (λ_max_ = 555 nm; see below), indicating the fluorophore is in a more hydrophobic lipid environment, showing specific interaction of α-syn with the membrane. Interestingly, slight variations of λ_max_ were observed, hinting at the existence of distinctive environments at individual locations. These results show that NBD is a useful reporter of α-syn localization and provides direct evidence of N-terminal α-syn binding to the inner leaflet of the plasma membrane.

### Site-Specific α-Syn–Lipid Interactions Revealed by NBD Fluorescence.

**Fig. 3.**
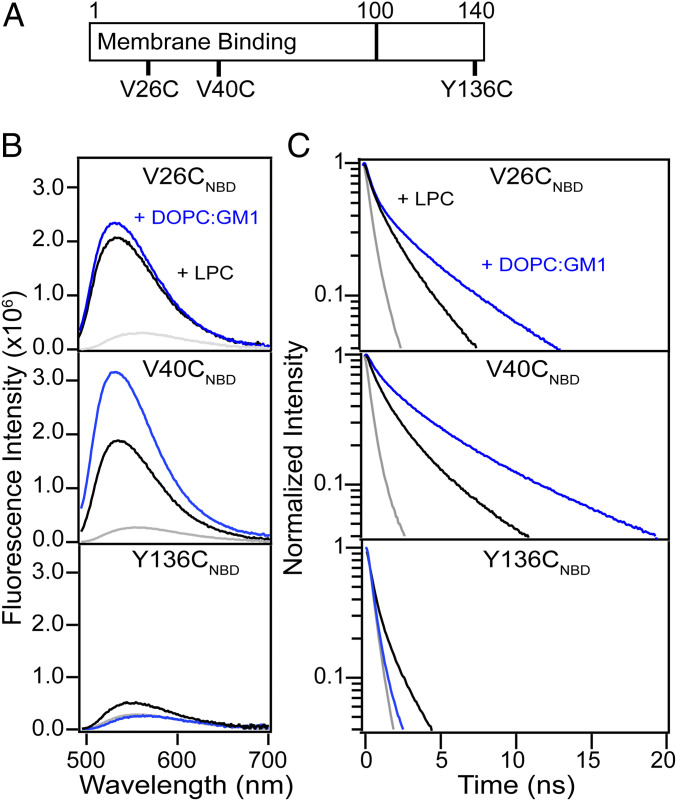
Site-specific NBD fluorescence of α-syn variants. (*A*) Schematic representation of the α-syn primary sequence. Membrane binding region (residues 1 to 100) and cysteine-labeling sites used in this study (V26C, V40C, and Y136C) are indicated. Comparison of steady-state fluorescence (*B*) and decay kinetics (*C*) of V26C_NBD_ (*Top*), V40C_NBD_ (*Middle*), and Y136C_NBD_ (*Bottom*) α-syn (1 µM) in buffer (gray) and in the presence of either LPC micelles (black; 5 mM) or DOPC/GM1 vesicles (blue; 1 mM). See *SI Appendix*, Tables S1 and S2 for fit parameters.

To develop a molecular understanding of how α**-**syn binds to biological membranes, conformational changes were evaluated using additional NBD variants (Fig. 3*A*). As a reference, we first characterized α-syn interactions with lysophosphatidylcholine (LPC) micelles and vesicles composed of DOPC/GM1. We chose PC because it is the most common lipid type in mammalian cells, and GM1 was selected because α-syn colocalized with GM1-containing areas on unroofed cells. Circular dichroism (CD) spectroscopy verified that all NBD variants adopted similar secondary structure as the wild-type protein in solution and in the presence of increasing micelles and vesicles with comparable binding affinities, indicating no effect by the NBD fluorophore (*SI Appendix*, Figs. S9*A* and S10).

In solution, all three NBD variants exhibited identical λ_max_ at 555 nm, characteristic of water-exposed fluorophores (Fig. 3*B* and *SI Appendix*, Fig. S9*B*). Both V26C_NBD_ and V40C_NBD_ spectra shifted to 533 nm in the presence of LPC micelles as the fluorophore partitions into the hydrocarbon acyl chains. Similarly, their spectra (λ_max_ = 530 nm) indicated NBD insertion into DOPC/GM1 vesicles, in accord with the elongated helical model (53). Interestingly, the spectrum of Y136C_NBD_ blue-shifted (λ_max_ = 542 nm) in the presence of LPC micelles, showing interaction of the acidic tail with zwitterionic lipids. This observation is consistent with a previous proposal that the glutamate-rich C-terminal region (residues 120 to 140) interacts with the positively charged choline head groups (54). In contrast, no discernible changes were measured in the presence of the negatively charged GM1 lipids due to electrostatic repulsion, indicating that interaction between the C-terminal tail and the membrane mimics is modulated by specific lipid composition and not driven by the fluorophore.

NBD decay kinetics revealed site-dependent differences (Fig. 3*C* and *SI Appendix*, Fig. S11). Decay kinetics were adequately fit to a double- or triple-exponential function, and the values are reported in the *SI Appendix*, Tables S1 and S2. For simplicity of discussion, only average fluorescence lifetimes (τ_avg_) are compared. As expected, all variants had relatively short and similar lifetimes in their disordered soluble states (τ_avg_ = 0.5 to 1.0 ns; *SI Appendix*, Fig. S9*C*). Upon micelle and vesicle binding, their lifetimes increased, reflecting a dramatic change in local environment and conformation. Interestingly, differences in the N-terminal region are seen, where position 40 has a higher sensitivity toward lipids (LPC: V40C_NBD_ [τ_avg_ = 3.8 ns] > V26C_NBD_ [τ_avg_ = 2.6 ns]; DOPC/GM1: V40C_NBD_ [τ_avg_ = 6.3 ns] > V26C_NBD_ [τ_avg_ = 4.8 ns]), which we attribute to differences in insertion depths and/or restricted motion when embedded in the lipid acyl chains. These results are in accord with the observed higher overall V40C_NBD_ emission intensity. The near twofold longer τ_avg_ observed for the N-terminal sites compared to that of micelles suggests a more rigid polypeptide conformation upon insertion into the lipid bilayer, similar to previously reported trends using Trp mutants, where N-terminal sites are sensitive probes of α-syn–lipid interactions (44). Consistent with the steady-state data, a longer lifetime for Y136C (τ_avg_ = 1.2 ns) was observed in the presence of LPC micelles, again indicating interaction of the C terminus with zwitterionic lipids. Together, our results demonstrate that NBD is an excellent site-specific probe of α-syn membrane interactions.

### Site-Specific Conformational Heterogeneity of α-Syn on Unroofed Cells.

**Fig. 4.**
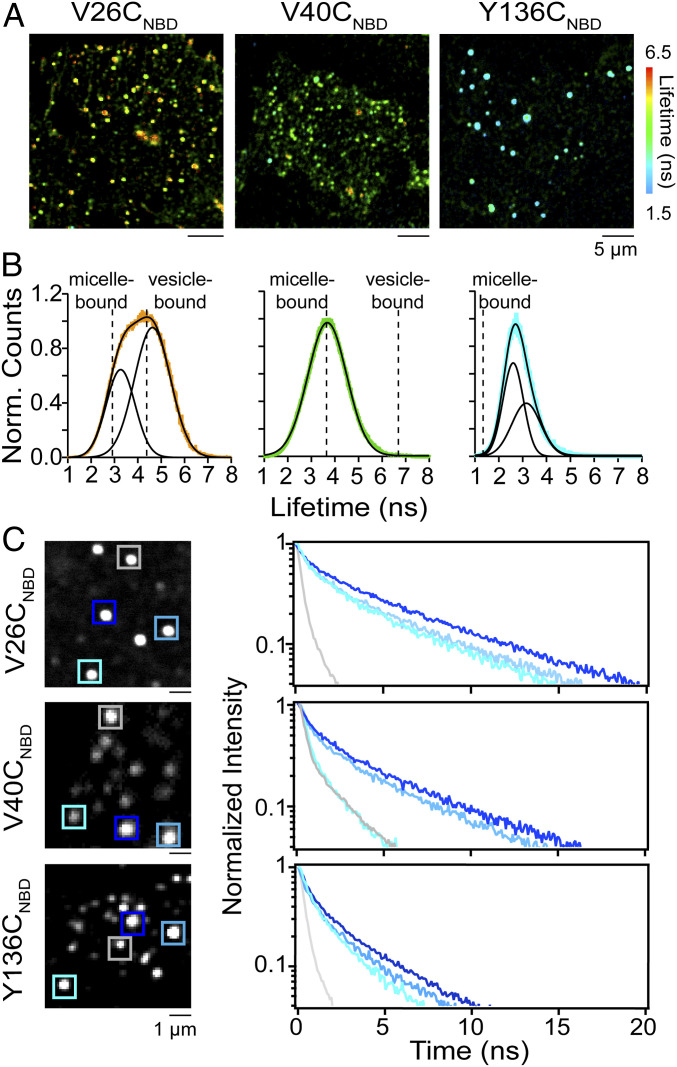
Site-specific α-syn conformational heterogeneity on unroofed cells revealed by FLIM. (*A*) Confocal fluorescence lifetime images of V26C_NBD_, V40C_NBD_, and Y136C_NBD_ α-syn on unroofed SK-MEL-28 cells. Red-to-cyan color scale spans lifetimes of 6.5 to 1.5 ns. (*B*) Fluorescence lifetime distributions of V26C_NBD_ (orange; 14,918 events from *n* = 46), V40C_NBD_ (green; 11,989 events from *n* = 30), and Y136C_NBD_ (cyan; 12,823 events from *n* = 35), where *n* represent the total number of unroofed cells from two biological replicates. The solid black lines represent Gaussian fits with χ^2^ values of 0.26 (V26C_NBD_), 0.17 (V40C_NBD_), and 0.24 (Y136C_NBD_). The dashed lines indicate τ_avg_ values obtained in the presence of LPC micelles and DOPC/GM1 vesicles. For comparison, single Gaussian fits for V26C_NBD_ and Y136C_NBD_ are shown in *SI Appendix*, Fig. S15. (*C*) NBD fluorescence decay kinetics of individual puncta for V26C_NBD_ (*Top*), V40C_NBD_ (*Middle*), and Y136C_NBD_ (*Bottom*). NBD decay kinetics were measured at locations indicated by colored boxes (*Left*). For fit parameters, see *SI Appendix*, Table S3. See *SI Appendix*, Figs. S12–S14 for additional lifetime images, distributions, and decay kinetics.

Since fluorescence decay kinetics (i.e., lifetimes) can reveal conformational differences of the membrane-bound α-syn populations that are not apparent from intensity-based analyses, we used fluorescence lifetime imaging microscopy (FLIM), a time-resolved image acquisition method to provide information on fluorescence lifetime distributions of NBD variants at different spatial locations on unroofed cells (Fig. 4*A*). Here, the contrast of the fluorescence image is based on the lifetime of individual fluorophores rather than their emission intensity.

Consistent with the intensity-based confocal fluorescence images (Fig. 1*C*), lifetime images of V26C_NBD_, V40C_NBD_, and Y136C_NBD_, displayed distinct punctate patterns on unroofed cells. However, each spot now exhibits a range of colors, representing the measured NBD lifetime per pixel from orange-to-green-to-cyan, spanning 5.5 to 2.5 ns (Fig. 4*A* and *SI Appendix*, Figs. S12–S14), where the τ_avg_ becomes shorter progressing from N-to-C sites, with the N terminus exhibiting the greatest sensitivity.

The fluorescence lifetime distributions (Fig. 4*B* and *SI Appendix*, Figs. S12–S14) of the three NBD-labeled variants indicate that each residue is in a hydrophobic environment as the lifetimes are longer than that measured for soluble α-syn (τ_avg_ = 0.5 to 1.0 ns). V26C_NBD_ exhibits the broadest lifetime distribution spanning from 2 to 7 ns, which is well described by two Gaussian functions with peak maxima centered at 3.2 and 4.6 ns, indicating at least two different conformers (Fig. 4 *B*, *Left*). While they are similar, the maxima do not align exactly with the measured τ_avg_ values of 2.6 and 4.8 ns for micelle- and vesicle-bound V26C_NBD_, respectively (Fig. 4 *B*, *Left*), suggesting that the conformational space that V26C_NBD_ samples is different from the two putative membrane-bound α-syn structures, the broken vs. elongated helix.

In contrast, the distribution of V40C_NBD_ fits well to a single Gaussian function centered at the micelle-bound τ_avg_ value of 3.8 ns (Fig. 4 *B*, *Middle*). Notably, V40C_NBD_ exhibits shorter lifetimes than V26C_NBD_, opposite to the trend characterized in either presence of lipid micelles or vesicles. Despite being only 14 residues apart, their behaviors are distinct on unroofed cells and not easily explained by the available structural models (9, 15, 55). For Y136C_NBD_, the lifetime distribution (Fig. 4 *B*, *Right*) indicates that the side chain is in a more hydrophobic environment, which contradicts current understanding, which presumes a disordered, water-exposed C-terminal tail. Unexpectedly, despite being the narrowest distribution, the distribution of Y136C_NBD_ is best fit with two Gaussian functions with peak maxima at 2.6 and 3.2 ns that are longer than that observed in the presence of micelles (Fig. 4 *B*, *Right*). This result implies that like V26C_NBD_, there are also at least two membrane-associated conformers of Y136C_NBD_.

The power of our coupled approach of using environmentally sensitive NBD and time-resolved fluorescence is reiterated by the comparison of images obtained through total intensity and the NBD decay kinetics measured at specified locations (Fig. 4*C* and *SI Appendix*, Figs. S12–S14). While spatially informative of the location of the protein, differences between individual spots are completely obscured in the intensity-based images (Fig. 4 *C*, *Left*). It is evident that NBD fluorescence decays vary at different puncta, indicative of variations of local environments, plausibly representing influences of specific lipids and/or proteins at exocytic areas (Fig. 4 *C*, *Right*). Moreover, while V26C_NBD_ is anticipated to exhibit heterogeneous behavior based on its broad distribution (Fig. 4*A*), both V40C_NBD_ and Y136C_NBD_ also experience complex environments as evidenced by the presence of long and short NBD fluorescence decay curves at different locations. Intriguingly, all three NBD fluorophores can be found in a water-exposed state as they exhibited short lifetimes at some puncta (Fig. 4*C*, gray curves; τ_avg_ = 0.7 to 1.6 ns; *SI Appendix*, Table S3), reminiscent of soluble α**-**syn. Additionally, Y136C_NBD_ is observed as having slower decays (Fig. 4*C*; blue curve; τ_avg_ = 2.7 ns), similar to those shown by V26C_NBD_ in the presence of LPC micelles. These observations, which are not apparent in the global distributions, are only revealed by careful measurements at the individual regional level. Although corresponding to rare events, it is clear that membrane-bound α-syn is much more conformationally dynamic than previously realized. This complexity with which α-syn interacts with lipid membranes is only captured through the use of cellularly derived, functionally relevant membranes.

## Discussion

The physiological function of α-syn remains poorly understood; however, membrane interactions are imperative to its proposed functions involving synaptic vesicle transmission and homeostasis. Here, we provide insights into the spatial localization of α**-**syn on the cytosolic membrane that is modulated by lipid/protein complexes pertinent to its function in exocytosis, revealing unexpected protein conformational behavior that challenges the current structural models of membrane-bound α-syn. By using unroofed cells, which encapsulate cellular membrane complexity and topology, we demonstrate that α-syn prefers binding to GM1-rich liquid-ordered lipid domains, which has not been documented before. As both GM1 and α-syn can stabilize and induce positive curvature in the plasma membrane, the spatial enrichment of both entities would prime the plasma membrane for vesicle fusion during exocytosis. As this process is largely driven by membrane curvature (31) and native protein–protein interactions that only exist on intact plasma membranes, recruitment of α-syn to these lipids would be abrogated in giant unilamellar vesicles (29), the prevalent model for evaluating protein partitioning between fluid and liquid-ordered phases. Clearly, simple membrane mimics are not sufficient as α-syn is proven to be extremely sensitive to a variety of membrane properties, including fluidity, curvature, packing defects, and surface charge.

α-Syn is reported to directly promote SNARE complex assembly (2, 56), and further, membrane-bound Rab3a can facilitate α-syn binding to synaptic vesicles (57). Both recruitment of specific lipids and presynaptic proteins such as α**-**syn may be necessary to either stabilize or induce membrane curvature (52), which needs to be tightly regulated during exocytosis. Conclusively, we show localization of α-syn with proteins (Rab3a, syntaxin-1A, and VAMP2) involved in exocytic vesicle docking and fusion, providing direct evidence of α-syn recruitment at exocytic vesicles on the inner plasma membrane.

Direct spatial observation of conformational heterogeneity of α-syn at the residue level on the cytoplasmic membrane was achieved by NBD lifetime imaging. Unexpectedly, V26C_NBD_ is more sensitive to the lipid environment compared to that of V40C_NBD_, which contradicts experiments performed with synthetic membrane models. In addition, V26C_NBD_ experiences the most heterogeneous environment of the sites investigated, which is counterintuitive as the N terminus is thought to be the membrane anchor, which would then be expected to adopt a well-folded, helical conformation. This inconsistency could be partly due to the fact that the structural models were determined using disordered lipids, which are disparate from the more ordered lipid environments at exocytic sites. Additionally, we reason that in order to accommodate the presence of numerous other proteins at the presynaptic plasma membrane, α-syn may only need to interact with lipids through fewer stretches of amino acids by adopting different local conformations as there are limited free areas for binding. Therefore, while the N-terminal region may have a high affinity for lipid membranes, the generalized α-helical conformation is perturbed by native biomolecules and could be modulated by specific protein–protein interactions. Because exocytic events are highly dynamic, involving coordination between both lipids and proteins, the equilibrium will shift between different α-syn conformations.

Generally, the C terminus of α-syn is thought to remain disordered in solution and thus minimally influences its membrane binding properties. However, our results presented here suggest otherwise. Data clearly indicate that the C terminus interacts with lipids, particularly with zwitterionic phospholipids, as indicated by the increase in fluorescence lifetime in the presence of LPC micelles. This observation is further substantiated on unroofed cells, suggesting that the C terminus could mediate lipid and/or protein interactions at the plasma membrane, related to its exocytic function.

Collectively, our results show that α-syn is more conformationally dynamic at the membrane interface than previously appreciated. We speculate that this inherent plasticity could also make the protein more susceptible to oligomerization at the membrane interface. It still remains unclear whether α-syn oligomerization plays a physiological role in assisting SNARE complex fusion as previously suggested (56) or potentially leads to the pathology of Parkinson’s disease by facilitating amyloid fibril formation (58, 59). Nevertheless, under disease-related conditions where α-syn accumulates in the cytosol, there would be an equilibrium shift and a reduction of its activity on the membrane, resulting in deterioration of neuronal function. Therefore, understanding α-syn conformational switching is of importance for future work; fluorescence resonance energy transfer experiments on unroofed cells (60) would be extremely informative in determining the conformational state of membrane-bound α-syn.

In conclusion, we show that α-syn prefers GM1-rich lipid domains and adopts a collection of diverse membrane-bound conformations in order to carry out its function at exocytic sites. These insights were only possible through the coupled approaches of using unroofed cells, derivatization of specific residues with the environmentally sensitive NBD fluorophore, and FLIM measurements. We demonstrate that unroofed cells are a suitable and powerful platform for spectroscopic studies that are broadly applicable. Our results with α-syn reaffirm the need to use cellularly derived and biologically relevant membranes for biophysical characterization in developing a molecular understanding of important protein–lipid interactions. We envision further investigations would provide insights into the conformational switching of α-syn from a physiological to pathological state and would be equally valuable if extended to other amyloidogenic and membrane-associated proteins.

## Materials and Methods

Details regarding reagents, proteins, cell culture, methods for immunofluorescence, confocal fluorescence microscopy, lipid vesicle preparation, fluorescence, and CD spectroscopy are provided in *SI Appendix*.

### Unroofing Cells.

Cells were unroofed following published protocols (34). Briefly, cells plated on #1.5 poly-d-lysine (PDL)-treated glass coverslips (Neuvitro; H-25–1.5-PDL) were transferred into stabilization buffer (30 mM Hepes, 70 mM KCl, 5 mM MgCl_2_, 3 mM EGTA, pH 7.4) prior to sonication. The sonicator tip (1/8″ tapered microtip; VWR International; 33996-163) was placed ∼4 to 5 mm above the coverslip, and one to two 400-ms pulses at the lowest power setting were applied (Branson Sonifier 450).

### Imaging Spectroscopy.

Unroofed cells were incubated with NBD-labeled α-syn for 5 min at room temperature, washed with stabilization buffer three times, and immediately after fixed in 2% paraformaldehyde for 20 min and stored at 4 °C. Unroofed cells were imaged using a 100× silicon oil-immersion objective (Olympus; UPLSAPO100XS) on an inverted microscope (Olympus; IX-73) equipped with an imaging spectrograph (Kymera 193i; Andor Oxford Instruments) with a 600 g/mm grating. The 488-nm line of an Ar-ion laser (Modu-Laser) was pass through a laser clean-up filter and directed to the sample using a 488-nm dichroic. To remove residual excitation light, a 488-nm long-passed emission filter was used. Each image and spectrum was acquired using 5-s exposure time and 10 accumulations with a slit width of 500 µm.

### FLIM Measurements.

Unroofed cells were prepared as described above. FLIM was carried out using a PicoQuant MicroTime 200 time-resolved confocal fluorescence microscope. The setup consists of an inverted Olympus IX-83 microscope equipped with a 60× water-immersion objective (Olympus; UPLSAPO60XW). NBD-labeled α-syn was excited using a 485-nm pulsed laser operating at a repetition rate of 16 MHz. The excitation light was guided to the sample by a 3-mm multiband-dichroic mirror 405/488. After passing a 100-µm pinhole and a 488-nm long-pass filter, the fluorescence was collected by a PMA Hybrid40 detector (PicoQuant). Images were collected with an integration time of 5 µs at each pixel. For soluble-, micelle- and vesicle-bound NBD-α-syn, time-correlated single-photon counting decays were acquired in point time trace mode with an average photon count greater than 10,000 counts. Images and decay curves were processed and fit using the SymPhoTime 64 software (PicoQuant).

## Supplementary Material

Supplementary File

## Data Availability

All data are made available within the article or *SI Appendix*.
