# Supplementary material for: Unroofing site-specific α-synuclein–lipid interactions at the plasma membrane
Source: Proc Natl Acad Sci U S A. 2020 Jul 27;117(32):18977–83. doi: 10.1073/pnas.2006291117 (PMC7430991; doi:10.1073/pnas.2006291117)
Supplement: Supplementary File [file pnas.2006291117.sapp.pdf]

Unroofing Site-Specific α-Synuclein Lipid Interactions at the Plasma Membrane

Upneet Kaur and Jennifer C. Lee

This SI Appendix includes:

Materials and Methods

Tables S1, S2, and **S3** Fluorescence lifetime fit parameters

Figure S1 Unroofing cell scheme and example confocal images at different z-positions comparing unroofed and intact cells

Figure S2 FRAP Data

Figure S3 Confocal fluorescence image of NBD control on unroofed cells

Figures S4, S5, and S6 Additional confocal fluorescence images

Figure S7 Pixel colocalization plots

Figure S8 Additional fluorescence imaging spectroscopy data

Figure S9 NBD labeled α-syn variants characterization in solution

Figure S10 CD binding curves

Figure S11 Additional NBD fluorescence decay kinetics

Figure S12, S13, and **S14** Additional fluorescence lifetime images

Figure S15 Gaussian fits for lifetime distributions

www.pnas.org/cgi/doi/10.1073/pnas.2006291117

Materials and Methods

Protein Expression and Purification. Recombinant N-terminally acetylated (Ac) α-syn (V26C, V40C, Y136C, and WT) were expressed and purified as previously published (1). One Shot BL21(DE3) cells (Thermo Fisher) were freshly co-transformed with pRK172 (2) and pNatB (3) plasmids and were grown in LB media (VWR) at 37 °C with shaking overnight (10 mL) and were used to inoculate 1-L TB media (VWR) supplemented with 0.4% glycerol. Once cells reached an $OD_{600\ nm}$ of ~ 0.8, the temperature was reduced to 30 °C, and protein expression was induced with 1 mM Isopropyl β-d-1-thiogalactopyranoside (IPTG) for ~16 h. Cell growth was under the selective pressure of carbenicillin (100 µg/mL) and chloramphenicol (35 µg/mL). For the purification of AcV26C-, AcV40C- and AcY136C-α-syn, 1 mM dithiothreitol (DTT) was supplemented in all buffers. Sample homogeneity and identity were evaluated using LC-ESI-MS (NHLBI Biochemistry Core) and SDS-PAGE visualized by silver staining (Pharmacia PhastGel 8–25%). All purified proteins were flash-frozen in liquid nitrogen and stored at −80 °C until use.

Protein Labeling. Stock solutions of the DyLight-488 maleimide (Thermo Fisher 46602) and IANBD Amide (*N*,*N*'-Dimethyl-*N*-(Iodoacetyl)-*N*'-(7-Nitrobenz-2-Oxa-1,3-Diazol-4-yl) Ethylenediamine, Thermo Fisher D2004) were prepared in dimethylformamide (DMF). To eliminate intermolecular disulfide bonds, 20 mM DTT was added to AcV26C-, AcV40C-, and AcY136C-α-syn at RT for 30 min prior to labeling. After the DTT treatment, the reduced protein was buffer exchanged into the reaction buffer (phosphate-buffered saline (PBS) and 1 mM EDTA at pH 7.4) using a PD-10 desalting column (GE Healthcare). A 3-molar excess of Dylight-488 maleimide or IANBD was then added to the protein solution ([α-syn] = 50 µM), and the reaction mixture was incubated for 2 h at RT in the dark. Afterwards, excess dye was removed by using a PD-10 desalting column. Concentration of labeled protein and degree of labeling were calculated using the following formulas:

$$[\text{DyLight-488}] = [A_{280\ nm} - (A_{493\ nm} \times 0.147)]/\ \varepsilon_{280\ nm}$$

$$\text{Degree of labeling} = A_{493\ nm}/(70{,}000 \times \text{protein conc.})$$

$$[\text{NBD}] = [A_{280\ nm} - (A_{470\ nm} \times 0.103)]/\ \varepsilon_{280\ nm}$$

$$\text{Degree of labeling} = A_{470\ nm}/(26{,}000 \times \text{protein conc.})$$

where the extinction coefficients were 5,970 $M^{-1}cm^{-1}$ for AcV26C and AcV40C and 4470 $M^{-1}cm^{-1}$ for AcY136C. Typical degree of labeling was 100% and molecular weight of the labeled protein was confirmed by LC-ESI-MS (NHLBI Biochemistry Core). $V26C_{NBD}$ and $V40C_{NBD}$ was measured to be 14,797.7 Da. $Y136C_{Dy488}$ and $Y136C_{NBD}$ were measured to be 15,219.7 and 14,765.23 Da, respectively.

Cell Culture. SK-MEL-28 (ATCC HTB-72) cells were maintained in phenol-free minimum essential medium (MEM, Thermo Fisher 41061307) supplemented with 10% fetal bovine serum (FBS, ATCC 30-2020) and 2% penicillin/streptomycin at 37 °C in 5% CO_2. SK-MEL-28 cells at 70–80% confluence in T-75 flasks were trypsinized with the addition of 0.25% Trypsin-EDTA (Thermo Fisher 25200056) for 2 min. Resuspended cells (1 mL) and fresh media were added to circular #1.5 glass coverslips coated with poly-D-lysine (Neuvitro H-25-1.5-PDL) in 6-well plates and allowed to grow for 24 h.

Fluorescent Labeling of Unroofed Cells. The lipid stains were prepared by diluting Vybrant DiD (Thermo Scientific V22889) and cholera toxin subunit-B (CT-B, Alexa-555 conjugate, Invitrogen C34776) into stabilization buffer (30 mM HEPES, 70 mM KCl, 5 mM $MgCl_2$, 3 mM EGTA, pH 7.4) to a final concentration of 2.5 and 1 ng/mL, respectively. Unroofed cells were allowed to incubate with each stain for 5 min at RT in the dark. After staining, the coverslips were rinsed with stabilization buffer three times to remove any excess dye. For confocal fluorescence microscopy experiments, appropriate amounts of dye-labeled α-syn were first diluted into stabilization buffer to a final concentration of 1 µM and added to the stained unroofed cells. These samples were imaged immediately in the presence of α-syn in solution without fixation.

Immunofluorescence. After unroofing, cells were incubated with appropriate concentrations of dye-labeled α-syn for 5 min, immediately after rinsed with stabilization buffer three times , and fixed in 2% PFA in stabilization buffer for 20 min. Coverslips were then rinsed with imaging buffer four times and blocked (3% bovine serum albumin (BSA)) for 1 h at RT. The cells were then labeled with primary antibodies (1h) for anti-Clathrin heavy chain (1:250, Abcam ab21679), anti-caveolin-1 antibody (1:250, Abcam ab17052), anti-Rab3a (1:200, Abcam ab3335), anti-syntaxin 1A (1:300, Abcam ab41453), anti-VAMP2 (1:200, Abcam ab3347), and anti-alpha synuclein (1:50, Abcam ab138501) diluted in blocking buffer. Coverslips were then washed with imaging buffer three times and stained for 1 h with the secondary antibody donkey anti-rabbit Alexa Fluor 647 (Thermo Fisher A-21235), diluted 1:1000 in blocking buffer. All procedures were conduct at RT. Coverslips were washed four times with imaging buffer and fixed in 2% PFA and stored at 4 °C.

Confocal Fluorescence Microscopy. Samples were imaged using a UPLSAPO 100×/1.35 NA silicone oil objective (Olympus, Tokyo, Japan) on an OlympusIX73 inverted microscope fitted with a Thorlabs Confocal Laser Scanner (CLS-SL) fiber coupled to a multichannel CMLS-E laser source. CT-B and Alexa Fluor 532 were excited using a 532 nm laser line and emission was collected using a 582 ± 75 nm bandpass filter. DiD and Alexa Fluor 642 were excited using a 642 nm laser line and emission was collected using a 660 nm long pass filter. NBD-labeled α-syn and $Y136C_{Dy488}$ α-syn were excited using a 488 nm laser line and emission was collected using a 512 ± 25 bandpass filter. A 50-µm pinhole was used and the scale was 0.1 µm/pixel. Images were analyzed with Fiji. Briefly, a Gaussian blur was applied to the raw image and the resulting image was used to subtract background signal. Next, the Otsu method was used to determine a threshold for binary images. Colocalized pixels were determined using Boolean Algebra ("intersection" function) to identify the pixels in which α-syn and the corresponding protein were both detected. Colocalization was analyzed by calculating a Pearson correlation coefficient (PCC) using the ImageJ plugin for colocalization (Coloc_2) for all acquired images. To verify the PCC values generated from Coloc2, object-based analysis was also used (ImageJ, JACoP). Multiple unroofed cells were imaged ($n \geq 50$) and trends were verified with at least two biological replicates and two independent treatments.

Fluorescence recovery after photobleaching (FRAP) experiments were acquired on a Zeiss 780 confocal microscope (NHLBI Light Microscopy Core) using a 63× oil immersion objective (NA 1.4). Fluorescence intensity recovery was monitored for approximately 120 s. CT-B fluorescence was excited by a 561-nm laser line and fluorescence was collected using a 605 ± 50 bandpass filter. DiD fluorescence was excited by a 633 nm laser line and fluorescence was collected using a 633

nm long pass filter. Photobleaching was performed using 100% laser power for each laser line for 50 iterations.

Phospholipid Micelle and Vesicle Preparation. Micelle stocks (100 mM) were produced by hydrating 16:0 LPC (Avanti, 855675P) in PBS buffer. Small unilamellar vesicles (SUVs) were prepared following previously established protocols (1). SUVs composed of 1,2-dioleoyl-sn-glycero-3-phosphocholine (DOPC; Avanti, 850375) and ganglioside GM1 (Avanti, 860065P) at a molar ratio of 4:1 were prepared by mixing of the different lipids in chloroform stock solutions. Lipids were dried under flowing nitrogen gas and to ensure removal of organic solvent the lipids were allowed to dry overnight at 50 °C in a vacuum oven. Lipid films were rehydrated in buffer (PBS, pH 7.4) and resuspended using bath sonication for 10 min (Branson 2510 Ultrasonic Cleaner). To produce SUVs, the resuspended lipids were probe-tip sonicated for 30 min in a water bath (Branson 450, output 6, 50% duty cycle). Vesicles were then centrifuged (17,000× g, 20 min) and syringe filtered through a 0.22 µm membrane (Millipore). Vesicle size (r ~ 40 nm) was determined by dynamic light scattering using a Wyatt Synapro NanoStar (NHLBI Biophysics Core). Micelles and SUVs were used immediately after preparation.

Fluorescence Spectroscopy. NBD-labeled protein (1 µM) was added to varying concentrations of 16:0 LPC and DOPC/GM1 vesicles. Emission was excited at 480 nm and monitored from 490 to 700 nm at 25 °C on a Fluorolog FL-3 instrument (Horiba Scientific) using 1-nm slit widths and 0.5 s integration times. All spectra were averages of 3 accumulations and their buffer/lipid backgrounds have been subtracted.

Circular Dichroism Spectroscopy. CD measurements were carried out on a JASCO J-715 instrument (NHLBI Biophysics Core) in a 1-mm quartz cuvette. Spectra were collected from 200–260 nm with the following settings: continuous mode, 1 nm steps, 100 nm/min, 1 nm bandwidth, 1 s integration time, and three accumulations at 20 °C. Buffer/lipid background subtractions were applied to all spectra and the mean residue ellipticity (MRE) was calculated using the following equation (4): where MRW represents the mean residue weight, θ_{deg} represents the ellipticity measured by the instrument, d and c represent the pathlength (cm) of the cuvette used and protein concentration (g/mL), respectively.

$$[\theta_{MRE}] = \frac{\theta_{deg}\, MRW}{10\, d\, c}$$

REFERENCES

1. O'Leary EI, Jiang Z, Strub MP, & Lee JC (2018) Effects of phosphatidylcholine membrane fluidity on the conformation and aggregation of N-terminally acetylated alpha-synuclein. *J Biol Chem* 293(28):11195-11205.
2. Jakes R, Spillantini MG, & Goedert M (1994) Identification of two distinct synucleins from human brain. *FEBS Lett* 345(1):27-32.
3. Johnson M, Coulton AT, Geeves MA, & Mulvihill DP (2010) Targeted amino-terminal acetylation of recombinant proteins in E. coli. *PLoS One* 5(12):e15801.
4. Kelly SM, Jess TJ, & Price NC (2005) How to study proteins by circular dichroism. *Biochim Biophys Acta* 1751(2):119-139.

Table S1. Fluorescence lifetime fit parameters of NBD-labeled Cys-variants of α-syn (1 μM) in the absence and presence of 5 mM Lyso-PC (16:0) micelles at pH 7.4[1]

	**[Micelle] (mM)**	$\boldsymbol{a_1}$	**τ_1 (ns)**	$\boldsymbol{a_2}$	**τ_2 (ns)**	**τ_{avg} (ns)**
V26C_{NBD}	0	0.26(1)	1.1(1)	0.74(1)	0.39(1)	0.73(5)
	5	0.6(2)	2.9(5)	0.4(1)	0.65(2)	2.55(3)
V40C_{NBD}	0	0.19(1)	1.6(14)	0.81(1)	0.42(1)	1.0(7)
	5	0.52(1)	4.5(2)	0.48(2)	1.2(1)	3.8(1)
Y136C_{NBD}	0	0.25(4)	0.9(3)	0.75(3)	0.41(2)	0.62(2)
	5	0.41(2)	1.5(1)	0.59(4)	0.4(5)	1.15(8)

[1] Data were fit to an exponential decay function: $I(t) = \sum_i a_i \exp\left(-t/\tau_i\right)$ using the SymPhoTime 64 software (PicoQuant) where a_i and τ_i are the pre-exponential and fluorescence lifetime, respectively. Uncertainty of the fit parameter is indicated. Instrument response ≤ 340 ps.

Table S2. Fluorescence lifetime fit parameters of NBD-labeled Cys-variants of α-syn (1 μM) in the absence and presence of increasing concentrations of 4:1 DOPC:GM1 vesicles (0–1 mM) at pH 7.4[1]

	**L:P**	a_1	**τ1 (ns)**	a_2	**τ 2 (ns)**	a_3	**τ 3 (ns)**	**τ avg (ns)**
V26CNBD	0	0.19(1)	1.10(1)	0.81(1)	0.39(1)			0.52(1)
	250	0.32(1)	5.6(2)	0.23(1)	1.8(2)	0.45(1)	0.45(2)	4.57(1)
	500	0.32(1)	5.6(2)	0.23(1)	1.8(2)	0.45(1)	0.45(2)	4.57(1)
	750	0.37(1)	5.7(1)	0.25(1)	1.91(7)	0.38(1)	0.46(2)	4.72(1)
	1000	0.36(1)	5.8(1)	0.26(1)	1.92(6)	0.38(1)	0.46(1)	4.77(4)
V40CNBD	0	0.38(2)	1.19(4)	0.62(2)	0.42(1)			0.72(1)
	250	0.38(2)	7.2(3)	0.33(1)	2.1(1)	0.29(1)	0.49(2)	5.7(1)
	500	0.39(1)	7.72(4)	0.39(1)	2.51(5)	0.22(1)	0.62(4)	5.9(1)
	750	0.35(1)	8.1(3)	0.39(2)	2.87(9)	0.26(2)	0.76(5)	6.24(2)
	1000	0.34(1)	8.1(1)	0.41(1)	2.63(5)	0.25(1)	0.63(2)	6.3(1)
Y136CNBD	0	0.40(4)	0.78(4)	0.60(4)	0.35(1)			0.52(1)
	250	0.15(1)	1.2(1)	0.85(1)	0.40(1)			0.55(5)
	500	0.21(1)	1.15(7)	0.79(1)	0.40(1)			0.56(1)
	750	0.26(1)	1.18(4)	0.74(1)	0.38(1)			0.59(1)
	1000	0.20(2)	1.4(1)	0.80(2)	0.40(1)			0.60(1)

[1] Data were fit to an exponential decay function: $I(t) = \sum_i a_i \exp\left(-t/\tau_i\right)$ using the SymPhoTime 64 software (PicoQuant) where a_i and τ_i are the pre-exponential and fluorescence lifetime, respectively. Uncertainty of the fit parameter is indicated. Instrument response ≤ 340 ps.

Table S3. Fluorescence lifetime it parameters of NBD-labeled Cys-variants of α-syn (1 µM) bound to unroofed cells[1]

	Decay Color	a_1	τ_1	a_2	τ_2	τ_{avg}
V26C$_{NBD}$	Grey	0.29(1)	1.24(1)	0.71(1)	0.29(2)	0.91(7)
	Cyan	0.70(1)	4.70(2)	0.30 (2)	0.59(3)	4.30(8)
	Blue	0.82(1)	5.2(1)	0.18(1)	0.51(1)	4.60(5)
	Navy	0.86(1)	5.4(2)	0.14(1)	0.54(2)	4.90(2)
V40C$_{NBD}$	Grey	0.29(4)	1.90(9)	0.71 (2)	0.41(2)	1.61(1)
	Cyan	0.28(1)	2.18(8)	0.72(1)	0.40(1)	1.87(2)
	Blue	0.61(4)	4.63(8)	0.39 (9)	0.53(1)	4.21(6)
	Navy	0.61(4)	5.10(8)	0.47(9)	0.53(1)	4.61(3)
Y136C$_{NBD}$	Grey	0.37(3)	0.9(1)	0.63(3)	0.20(2)	0.71(1)
	Cyan	0.32(2)	2.46(3)	0.68(1)	0.43(1)	2.14(1)
	Blue	0.45(1)	2.91(5)	0.55(1)	0.45(2)	2.57(3)
	Navy	0.55(2)	3.03(1)	0.45(2)	0.54(4)	2.71(6)

[1] Data were fit to an exponential decay function: $I(t) = \sum_i a_i \exp\left(^{-t}/_{\tau_i}\right)$ using the SymPhoTime 64 software (PicoQuant) where a_i and τ_i are the pre-exponential and fluorescence lifetime, respectively. Uncertainty of the fit parameter is indicated. Instrument response ≤ 340 ps.

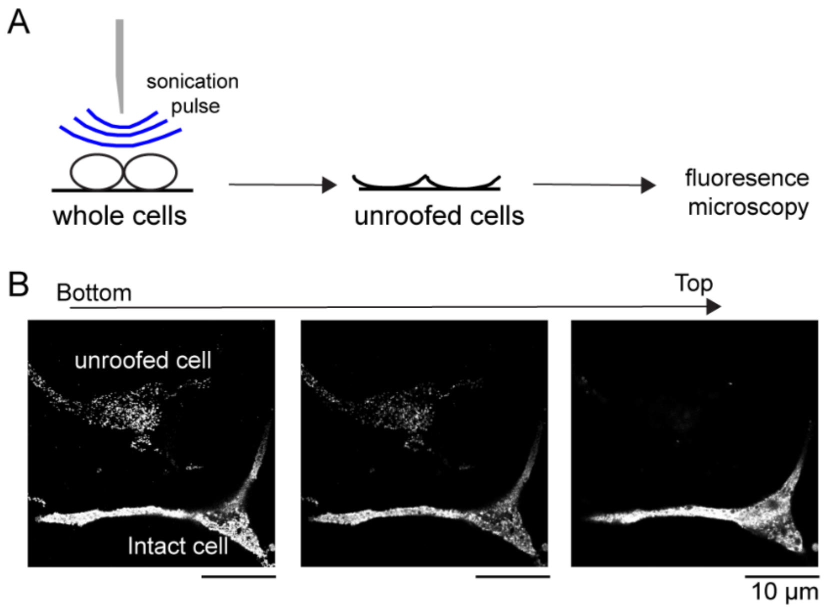

Figure S1. (A) Schematic of cellular unroofing. Cultured cells are exposed to a brief sonication pulse which disrupts cellular contents leaving behind the basal plasma membrane. (B) Confocal fluorescence *z*-stack images (*z* step-size = 0.2 μm) of SK-MEL-28 cells immunostained for clathrin-coated structures. The field of view contains an intact and unroofed cell as labeled.

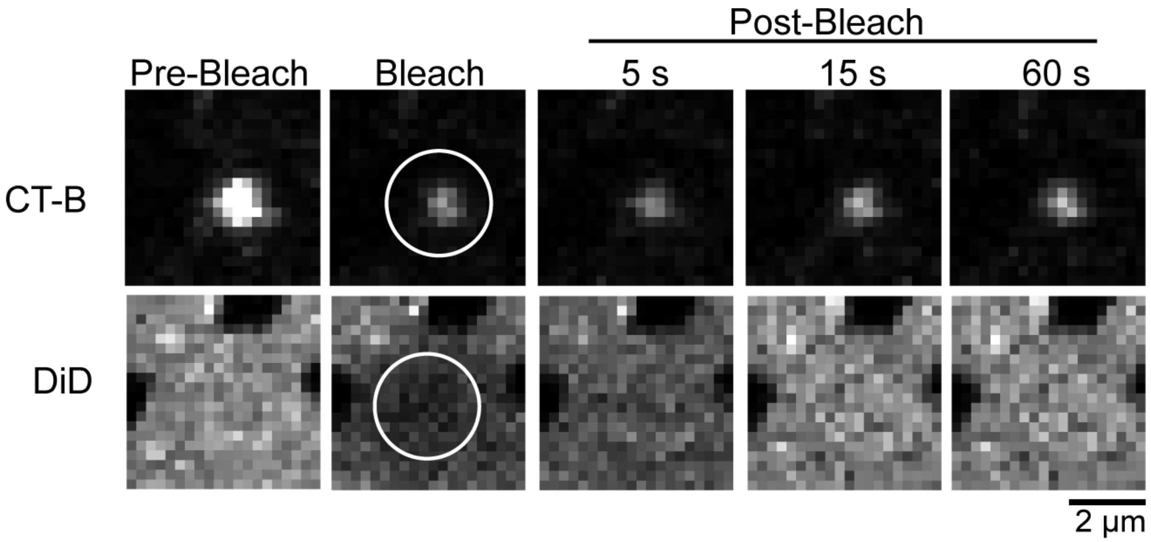

Figure S2. Membrane fluidity of unroofed cells measured by fluoresce recovery after photobleaching (FRAP) experiments**.** Representative CT-B and DiD stained unroofed cells were bleached in a circular region of interest (ROI; indicated by white circle) and fluorescence recovery was monitored post-bleaching.

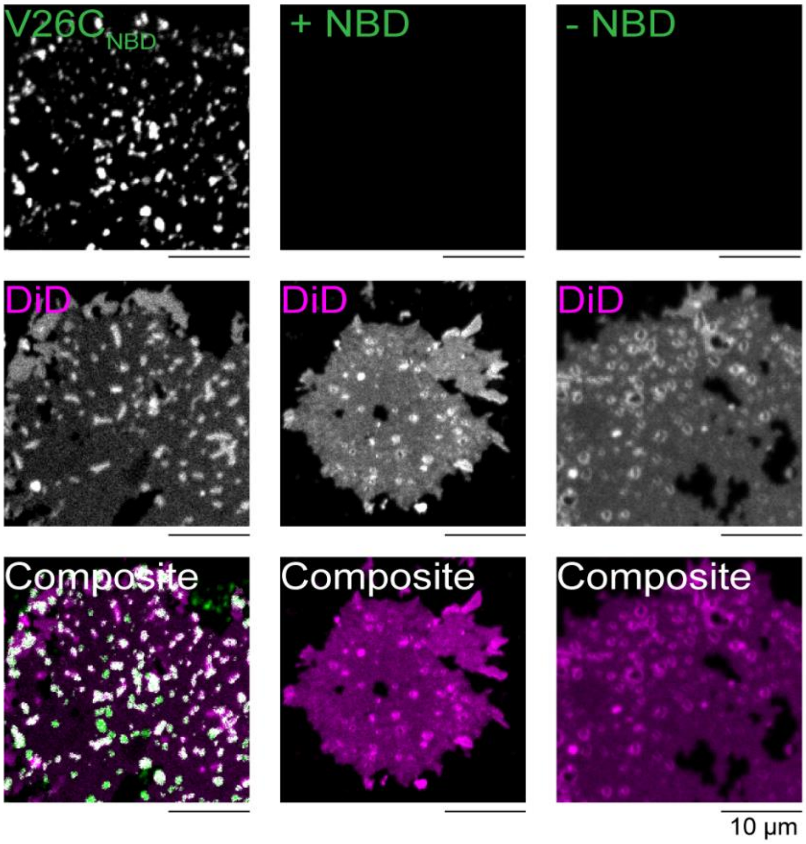

Figure S3. Representative confocal fluorescence images of $V26C_{NBD}$ α-syn (green), NBD fluorophore (+NBD, green), and a negative control (-NBD, green) on unroofed cells stained with DiD (magenta). Colocalized areas appear in white in the composite images. Scale bars are 10 µm.

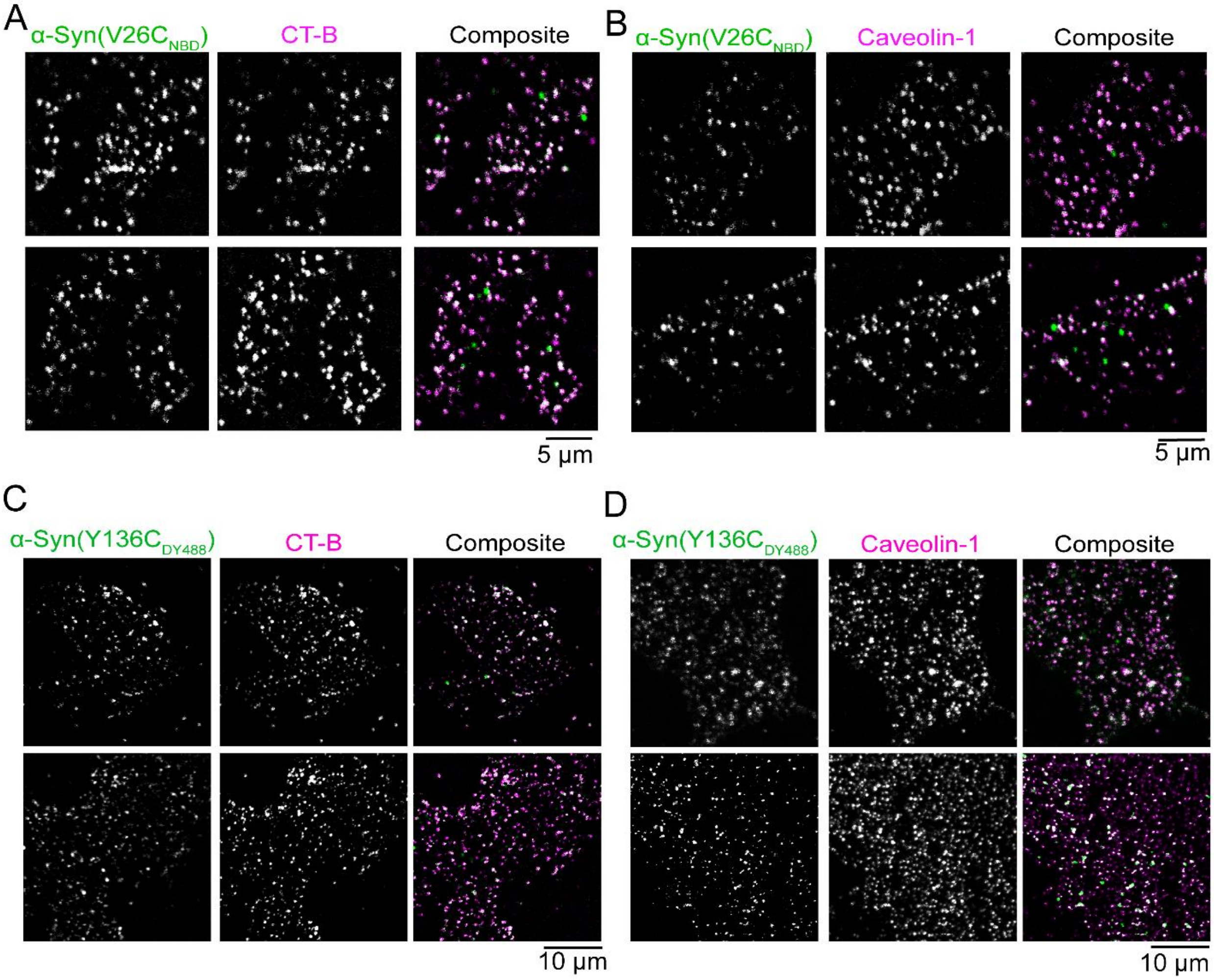

Figure S4. Confocal fluorescence images of $V26C_{NBD}$ α-syn (green) colocalization with (A) CT-B (magenta) and (B) caveolin-1 (magenta) and of $Y136C_{Dy488}$ α-syn (green) colocalization with (C) CT-B (magenta) and (D) caveolin-1 (magenta). Colocalized areas appear in white in the composite images.

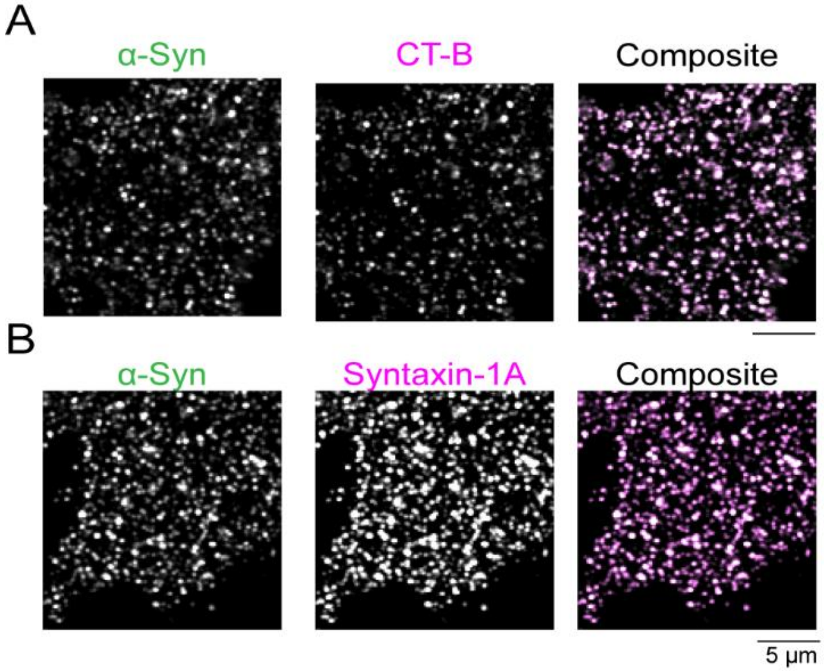

Figure S5. Confocal fluorescence images endogenous α-syn (green) colocalization with (A) CT-B (magenta), (B) Syntaxin-1A (magenta). Colocalized areas appear in white in the composite images.

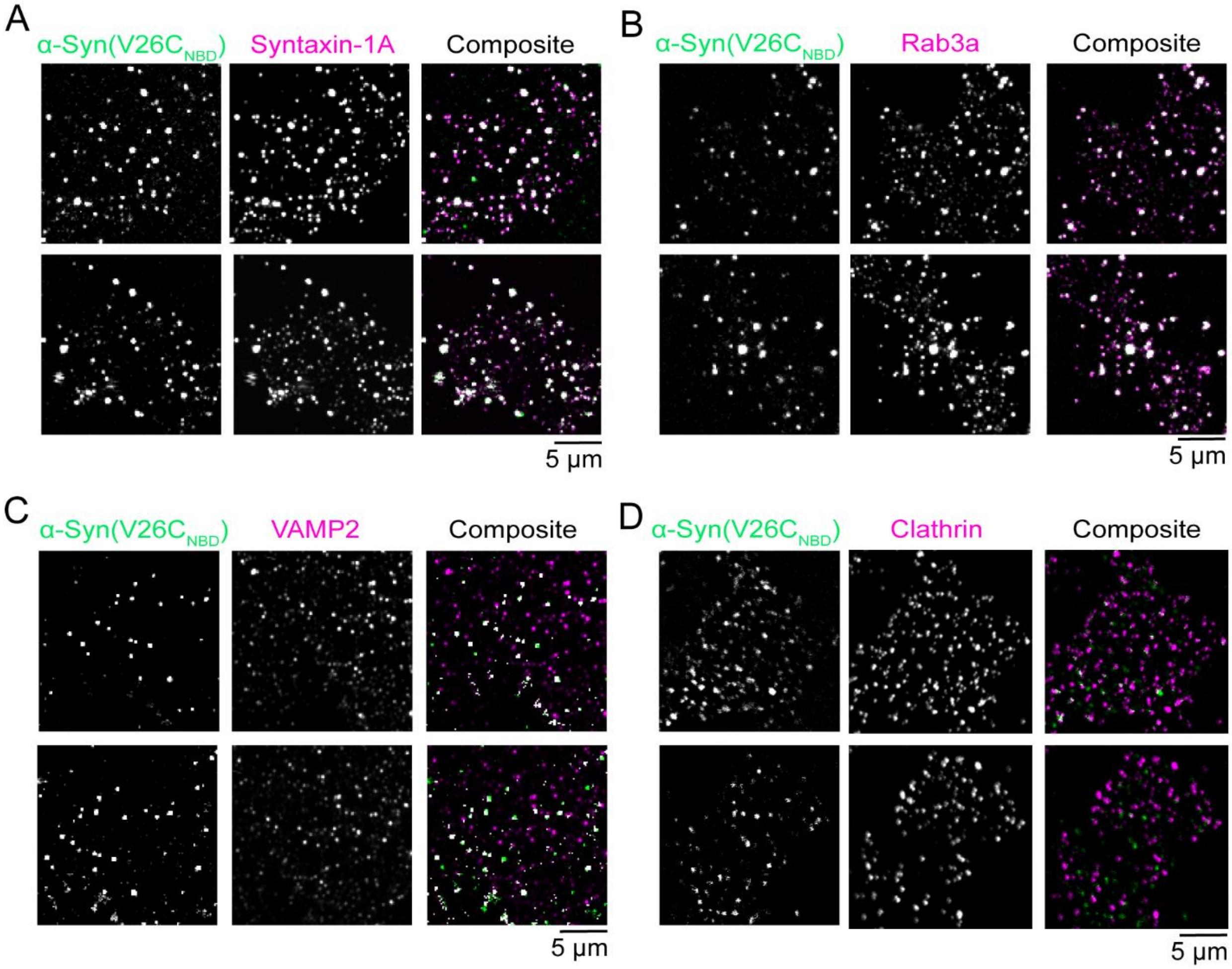

Figure S6. Confocal fluorescence images V26C$_{NBD}$ α-syn (green) colocalization with (A) syntaxin-1A (magenta), (B) Rab3a (magenta), (C) VAMP2 (magenta), and (D) Clathrin (magenta). Colocalized areas appear in white in the composite images.

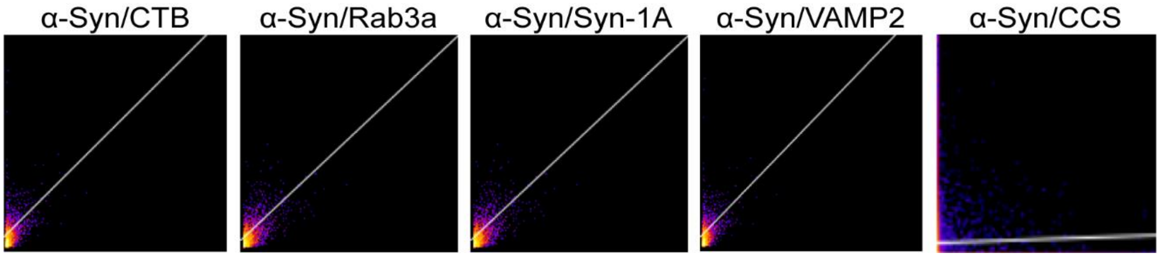

Figure S7. Representative pixel plots generated by ImageJ Coloc2 for colocalization analysis of V26C$_{NBD}$ α-syn with either CT-B, Rab3a, Syn-1A, VAMP2, or CCS. The plots are specifically for the images shown in **Figure 1**.

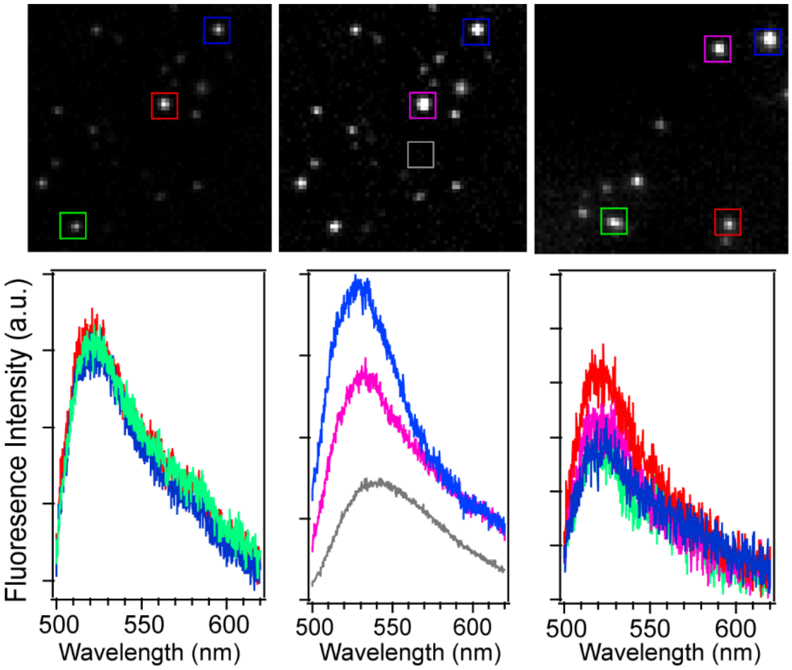

Figure S8. Additional spatially-resolved spectra of V26C$_{NBD}$ α-syn bound to unroofed cells. Widefield fluorescence images of α-syn (V26C$_{NBD}$) on unroofed SK-MEL-28 cells (top) and fluorescence spectra taken at spatial locations indicated by the colored boxes (bottom).

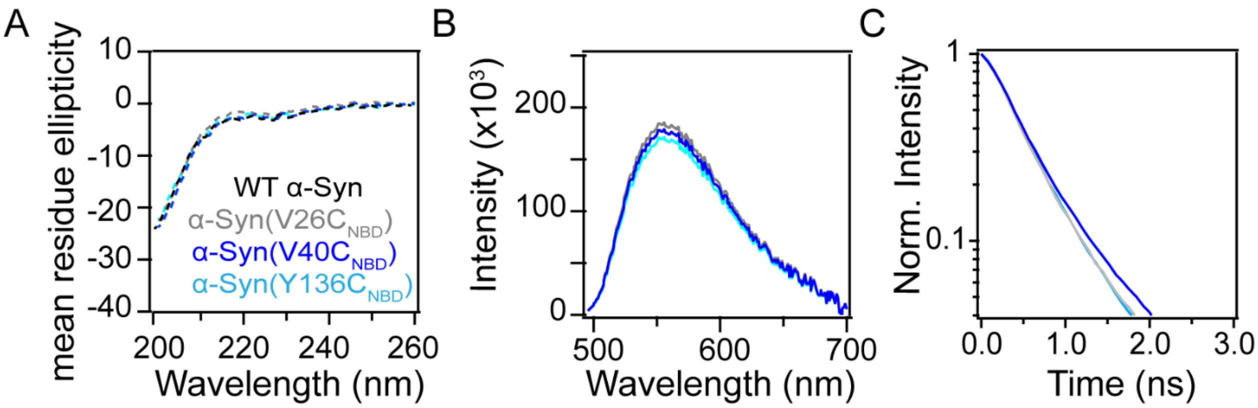

Figure S9. NBD-labeled α-syn variants characterization in solution. (A) CD spectra, (B) fluorescence steady-state spectra, and (C) fluorescence lifetime decays (right panel) are shown for V26C$_{NBD}$ (gray), V40C$_{NBD}$ (blue), and Y136C$_{NBD}$ (cyan).

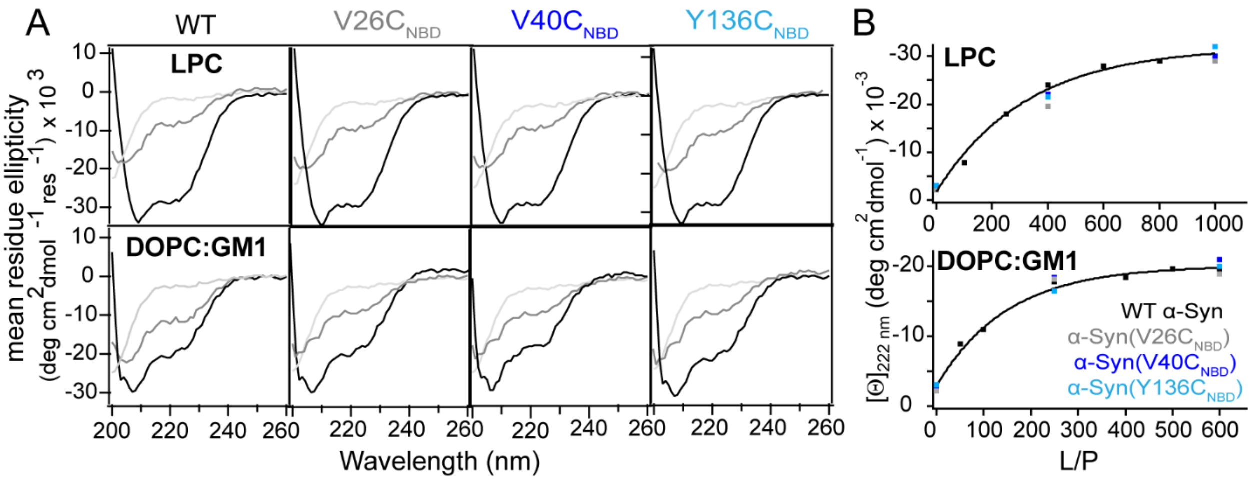

Figure S10. Secondary structure characterization of α-syn with lipid micelles and vesicles (A) CD measurements of WT, V26C$_{NBD}$, V40C$_{NBD}$, and Y136C$_{NBD}$ α-syn with 16:0 lyso-PC (LPC) micelles at L/P ratios of 0, 400, and 1000 ([protein] = 5 μM, [LPC] = 25 mM) and DOPC/GM1 SUVs at L/P ratios of 0, 250, and 600 are shown ([protein] = 5 μM, [DOPC:GM1] = 5 mM). (B) Binding curves from mean residue ellipticity ([θ_{222nm}) for WT α-synuclein (black) with LPC micelles (top plot) and DOPC:GM1 vesicles (bottom plot) where fits are shown with a solid black line. MRE values of NBD-labeled variants V26C (grey), V40C (blue), and Y136C (cyan) at L/P ratios of 0, 400, and 1000 are shown for LPC micelles. MRE values of NBD-labeled variants V26C (gray), V40C (blue), and Y136C (cyan) at L/P ratios of 0, 250, and 600 are shown for DOPC:GM1 vesicles.

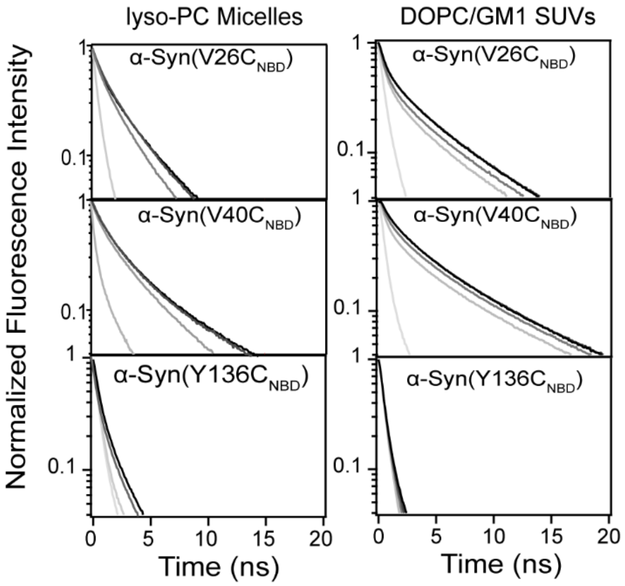

Figure S11. Site-specific NBD fluorescence decay kinetics of α-syn variants in buffer (gray) and in the presence of increasing concentrations of either LPC micelles or DOPC/GM1 vesicles. Concentrations of α-syn, LPC, and DOPC:GM1 were 1 µM and 0–25 mM, and 0–1 mM, respectively.

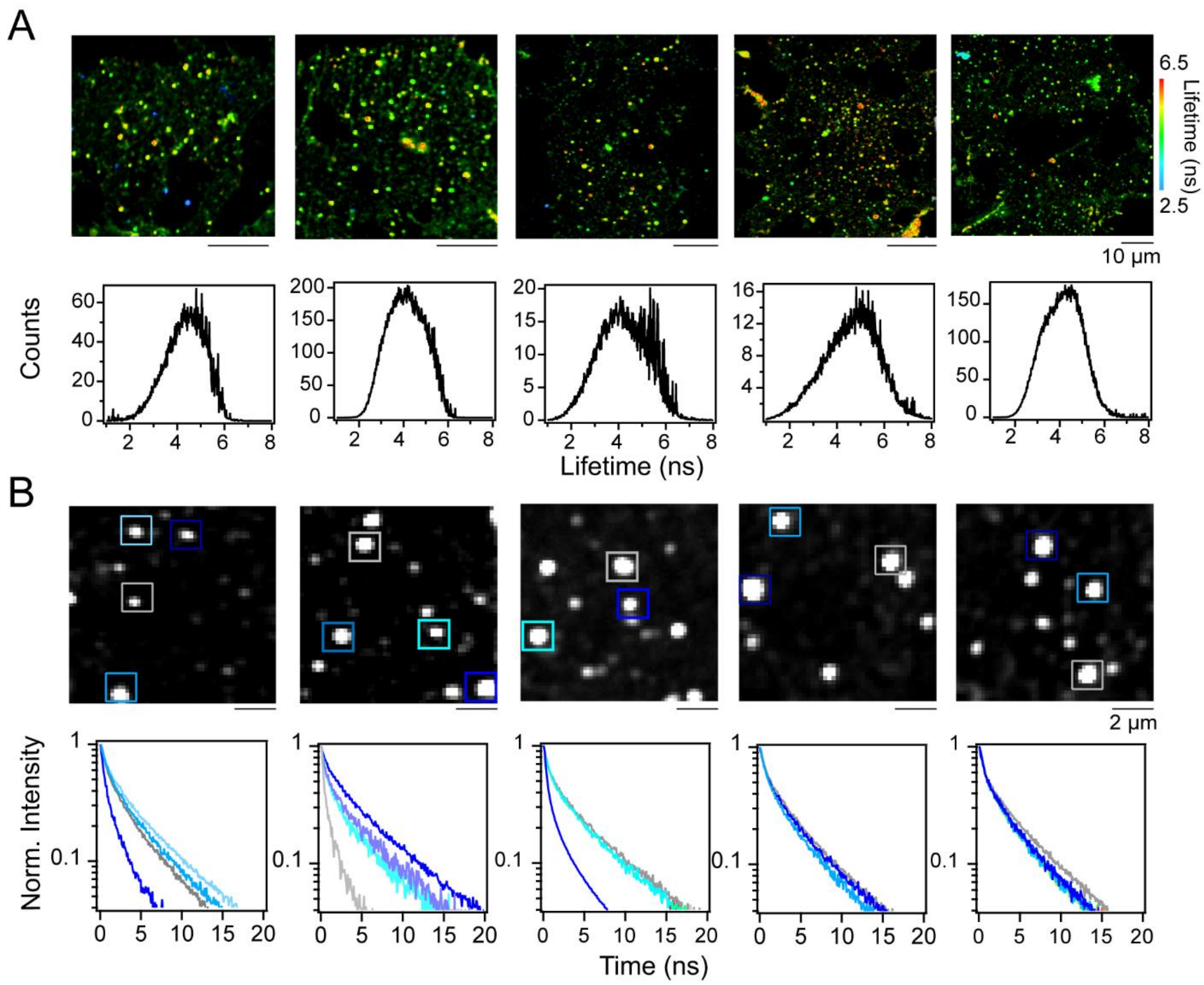

Figure S12. (A) Confocal fluorescence lifetime images of α-syn (V26C$_{NBD}$) on unroofed cells (top) and corresponding average lifetime distributions histograms for each image (bottom). Red-to-cyan color scale spans lifetimes of 6.5 to 2.5 ns. (B) NBD fluorescence decay kinetics of individual puncta for α-syn V26C$_{NBD}$ unroofed cells. Colored traces were measured at locations indicated by colored boxes.

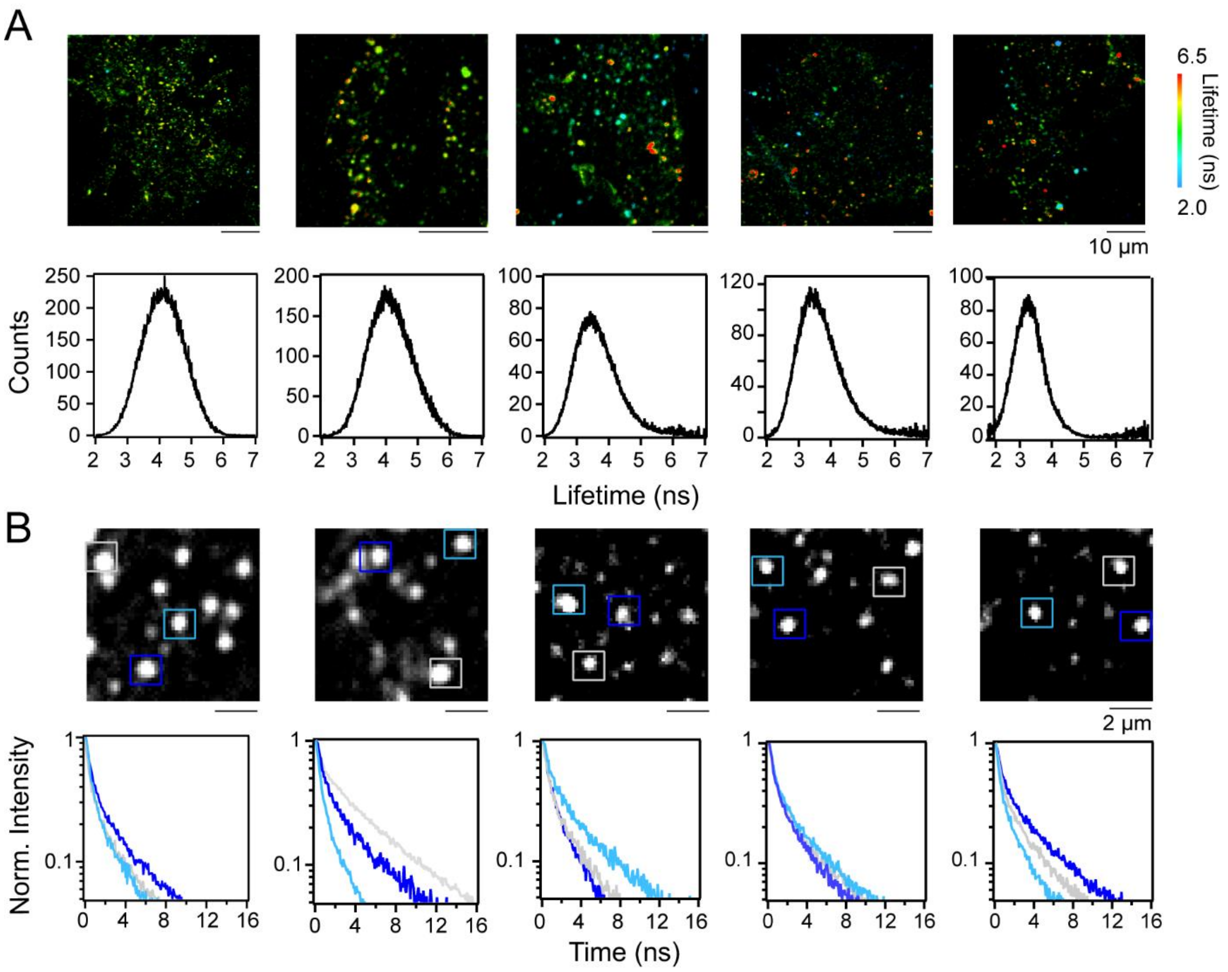

Figure S13. (A) Confocal fluorescence lifetime images of α-syn ($V40C_{NBD}$) on unroofed cells (top) and corresponding average lifetime distributions histograms for each image (bottom). Red-to-cyan color scale spans lifetimes of 6.5 to 2.0 ns. (B) NBD fluorescence decay kinetics of individual puncta for α-syn $V26C_{NBD}$ unroofed cells. Colored traces were measured at locations indicated by colored boxes.

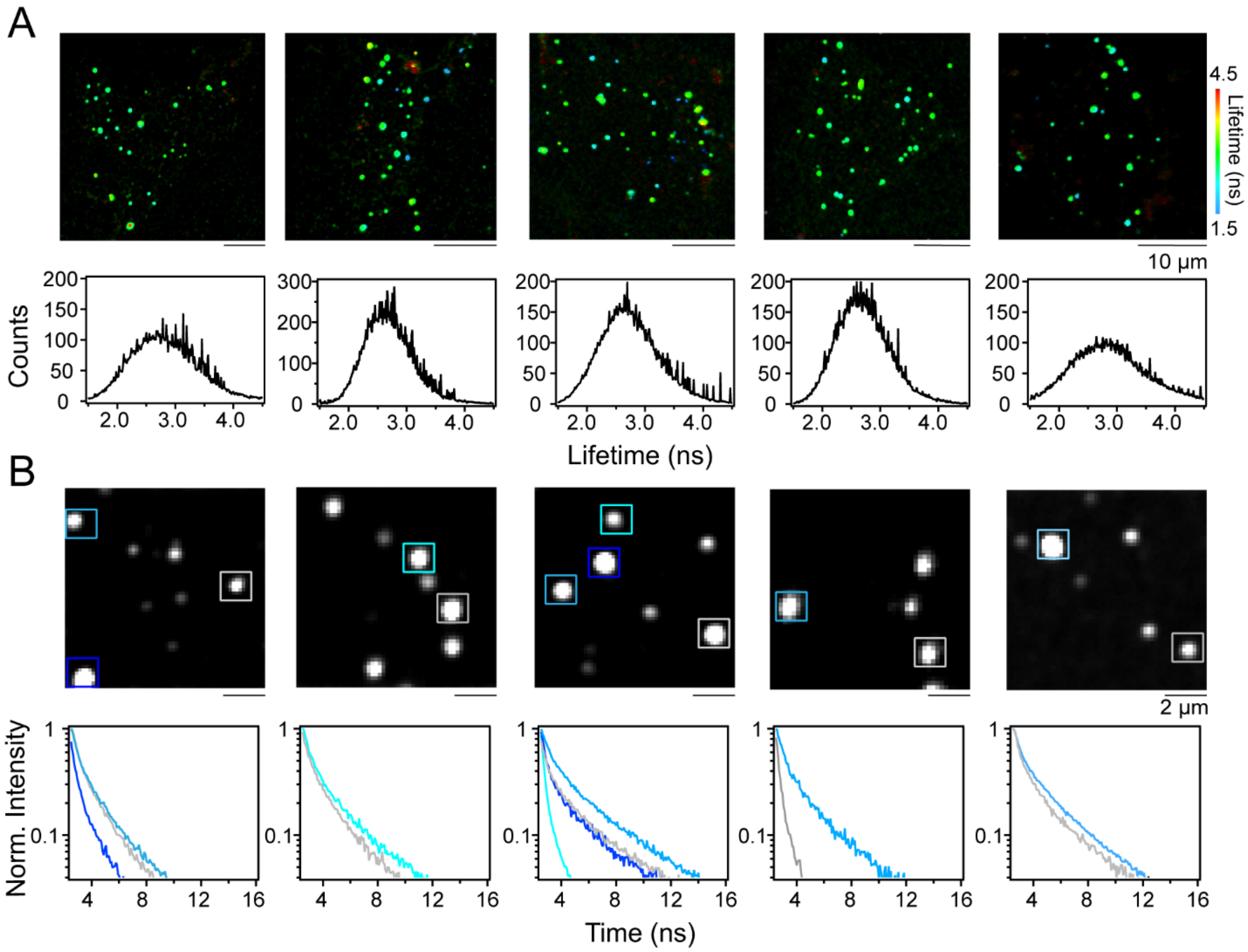

Figure S14. (A) Confocal fluorescence lifetime images of α-syn (Y136C$_{NBD}$) on unroofed cells (top) and corresponding average lifetime distributions histograms for each image (bottom). Red-to-cyan color scale spans lifetimes of 4.5 to 1.5 ns. (B) NBD fluorescence decay kinetics of individual punctates for α-syn V26C$_{NBD}$ unroofed cells. Colored traces were measured at locations indicated by colored boxes.

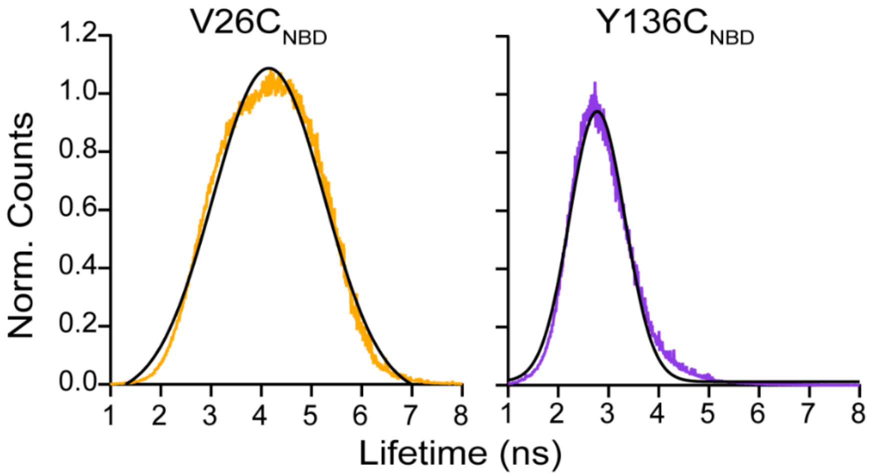

Figure S15. Fluorescence distributions of V26C$_{NBD}$ and Y136C$_{NBD}$ as shown in Fig.4B. Solid black line represents fit to a single gaussian fit resulting in χ^2 values of 1.95 and 1.04 for V26C$_{NBD}$ and Y136C$_{NBD}$, respectively.
